# Combining anti-CTLA4 with RG7787, an immunotoxin targeting mesothelin, promotes tumor eradication

**DOI:** 10.1186/2051-1426-3-S2-P190

**Published:** 2015-11-04

**Authors:** Jasmin Leshem, Xiu-fen Liu, Tapan Bera, Masaki Terabe, Jay A Berzofsky, Birgit Bossenmaier, Gerhard Niederfellner, Ira Pastan

**Affiliations:** 1 Laboratory of Molecular Biology, National Cancer Institute, Bethesda, MD, USA; 2 National Cancer Institute, Bethesda, MD, USA; 3 NCI/CCR/Vaccine Branch, Bethesda, MD, USA; 4 Roche Pharmaceutical Research &Early Development, Discovery Oncology, Innovation Center Penzberg, Roche Diagnostics GmbH, Penzberg, Germany

## Introduction

Antibodies to cytotoxic T-lymphocyte-associated protein 4 (CTLA4) potentiate an immune response against cancer. Response rates in melanoma patients are range from 10 to 19%[1]. To increase the efficacy and variety of cancer types treated by anti CTLA4, a combination therapy should be pursued. RG7787 is a recombinant immunotoxin composed of an anti-mesothelin Fab fused to a fragment of Pseudomonas exotoxin A (PE). RG7787 is currently tested in phase 1 trials for mesothelin expressing malignancies. We previously observed that an anti-mesothelin immunotoxin produced major tumor regressions in humans with advanced mesothelioma when combined with Cytoxan and Pentostatin apparently due to T cell activation[2] and we are exploring other ways to activate the immune system when cells are killed by immunotoxin. We hypothesize that combining RG7787 with anti CTLA4 will promote activation of the immune system and cancer elimination.

## Methods

We transfected the 66C14 mouse breast cancer cell line with human mesothelin and grew the cancer cells in immune-competent BALB/c mice that express a human mesothelin transgene so that they will not reject the tumors. The transfected cells are killed by RG7787 in cell culture (*IC_50_* 12 ng/ml). Anti-tumor activity was evaluated by intra tumoral-injection of RG7787 combined with intra-peritoneal injection of anti CTLA4.

## Results

We found that combining RG7787 with anti CTLA4 produced a 76% complete remission (CR) rate, while CR was reached in only 15% of the mice under anti CTLA4 monotherapy. The survival benefit was statistically significantly (P= 0.0012). No mice reached CR after treatment with RG7787 alone or in the PBS treated controls. Furthermore, re-challenging of the mice that reached CR with the parental cell line (66C14 without human mesothelin) resulted in complete rejection of the tumor in all the mice. We also found treatment with antibodies to CD8 decreased the CR rate to 12%, indicating that CD8^+^ T cells are necessary for the response.

## Conclusions

Combining RG7787 with anti CTLA4 produces a high rate of complete remissions in a breast cancer model.
